# Effective beam method for element concentrations

**DOI:** 10.1107/S1600577514026575

**Published:** 2015-01-29

**Authors:** Thomas Tolhurst, Mauricio Barbi, Tim Tokaryk

**Affiliations:** a University of Regina, Canada; b Royal Saskatchewan Museum, Canada

**Keywords:** beam method, element concentration

## Abstract

There is a great diversity of research being conducted at synchrotron facilities around the world and a diverse set of beamlines to accommodate this research. Time is a precious commodity at synchrotron facilities; therefore, methods that can maximize the time spent collecting data are of value. At the same time the incident radiation spectrum, necessary for some research, may not be known on a given beamline. A preliminary presentation of a method applicable to X-ray fluorescence spectrocopic analyses that overcomes the lack of information about the incident beam spectrum that addresses both of these concerns is given here. The method is equally applicable for other X-ray sources so long as local conditions are considered. It relies on replacing the polychromatic spectrum in a standard fundamental parameters analysis with a set of effective monochromatic photon beams. A beam is associated with each element and can be described by an analytical function allowing extension to elements not included in the necessary calibration measurement(s).

## Abstract 2

A method to evaluate chemical element concentrations in samples by generating an effective polychromatic beam using as initial input real monochromatic beam data is presented.

## 1. Introduction   

A collaboration between the University of Regina and the Royal Saskatchewan Museum (the Integrated Palaeontology Working Group) is conducting research to probe fossils at microscopic level using the synchrotron radiation facilities available at the Canadian Light Source in Saskatoon, Saskatchewan. This research is focused on the qualitative and the quantitative analysis of fossil chemistry in the pursuit of palaeo-ecological information. An important part of this work is the use of hard X-rays for fluorescence (XRF) analysis. There are pre-existing packages for the analysis of X-ray fluorescence spectra (Solé *et al.*, 2007 ▶), but they often require knowledge of the incident beam spectrum. One of the primary beamlines (VESPERS – very sensitive elemental and structural probe employing radiation from a synchrotron; Feng *et al.*, 2009 ▶) used by the research group does not have this spectrum readily available. Further, as the endstation is not under vacuum, the exact spectrum that would interact with the sample is not straightforward to measure due to attenuation effects. In addition, if a low-*Z* material is used to measure this spectrum, the result would be an even further attenuated incident beam due to scattering effects. Placing a detector at the sample position itself may be complicated due to a high count rate. In any case, all of this would imply the use of valuable beam time, especially if new electronics are to be introduced or the beamline set-up significantly changed in order to allow the measurement of the incident beam spectrum. On the other hand, the method proposed here requires nothing more than a brief (∼1 min) XRF measurement on the VESPERS beamline to assess the incident spectrum without changing any aspect of the beamline set-up.

## 2. Effective beam   

To determine element concentrations with a fundamental parameters method (Bertin, 1978 ▶; Beckhoff *et al.*, 2006 ▶), the energy and relative intensity of all incident photons must be known; that is, the incident beam spectrum. With a high-energy-resolution monochromatic beam [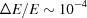 on VESPERS (Feng *et al.*, 2009 ▶)], this is not essential, but is essential for a polychromatic beam.

The intensity of an emission line resulting from the excitations of atoms of a given species in a sample is directly related to the concentration of those atoms in that sample. A given emission line resulting from atomic excitations by a polychromatic beam can always be replicated by an effective monochromatic beam. By adjusting the intensity and/or energy of the monochromatic beam, the same result as for the polychromatic beam can be achieved (Bertin, 1978 ▶). In the case of a polyatomic sample, with multiple emission lines of interest, there is an additional complication to contend with. An effective monochromatic beam that replicates the effect of the polychromatic beam for one element will not necessarily work for another element in the sample. For the case with *N* atoms in a sample, there is no guarantee that a monochromatic beam can be found that will replicate the effect of the polychromatic beam for all elements present in this sample. A solution is to construct an ‘effective polychromatic beam’ composed of *N* perfectly monochromatic beams. Each of the *N* beams would mostly affect a given atom species in the sample depending on the cross section for the photon–atom inter­action at a given energy. In theory, such a polychromatic beam can always be found.

One way to develop an effective polychromatic beam is by modifying the standard fundamental parameters equation, as presented by Beckhoff *et al.* (2006 ▶), for primary and secondary excitation in an infinitely thick sample, such that
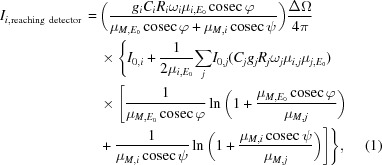
where *i* is used to denote all the relevant aspects of an element leading to the emission line of interest from that element. 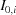 is the *i*th effective monochromatic beam, of energy 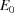, associated with and affecting predominantly the *i*th element. 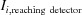 is the detected intensity of element *i* (assuming 100% intrinsic efficiency). The incident and detection angles are ϕ and ψ, respectively. The variables 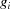, 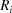 and 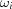 are, respectively, the edge jump ratio, the branching ratio and the fluorescence yield for element *i*, which lead to the emission of the line of interest. 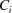 is the weight percent of the *i*th element. 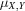 are mass absorption coefficients for a material *X* at photon energy *Y*. The matrix is denoted as *M* and single elements denoted with *i* or *j*. When 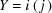, the implication is that it is a characteristic line of 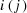. The photodetector solid angle is included and written as 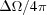. If the set of *N* beams is known, the concentration of the *i*th element can be determined through an iterative method.

## 3. Reference sample   

To properly determine the effective beam, a standard is needed. The standard must have known concentrations of several elements. Here the standard used is SRM 610 from the National Institute of Standards and Technology (NIST) (Wise & Watters, 2012 ▶). Any sample with a set of known element concentrations could be used, though ideally it should be similar in its properties to future analytes. SRM 610 is a glass matrix impregnated with 61 elements with concentrations ranging from 100 to 500 mg kg^−1^. The standard is a 3 mm-thick circular wafer with a diameter of ∼13 mm.

The element concentrations (mass fractions) in the standard are guaranteed to varying degrees by the manufacturer. There are 15 certified elements, whose concentrations are given with a high degree of confidence. Two reference values are given. These are not certified, but are best estimates which do not meet the certification criteria. Information values are given for seven elements. These are values considered of interest, but with uncertainties that were not able to be determined (May *et al.*, 2000 ▶). In this analysis, the uncertainty of the information value mass fractions are taken at twice the mean uncertainty (σ_sd_) of the certified and reference values. A systematic error based on this assumption is included, taken to be one half of the difference between the results computed assuming an uncertainty of 1σ_sd_ and 3σ_sd_ for the information values.

The elements composing the glass matrix are not included in this classification. The matrix is specified as having mass fractions: 72% SiO_2_, 14% Na_2_O, 12% CaO and 2% Al_2_O_3_. Errors for these concentrations are assigned at 2σ_sd_. The sample is manufactured to give ≤2% relative repeatability with the illumination of the full wafer. The data for this analysis were collected using a beam spot (diameter) ≤10 µm. Some deviations from the nominal concentrations can be expected at this small scale.

## 4. Results   

### 4.1. Effective beam calibration   

**Figure 1:**
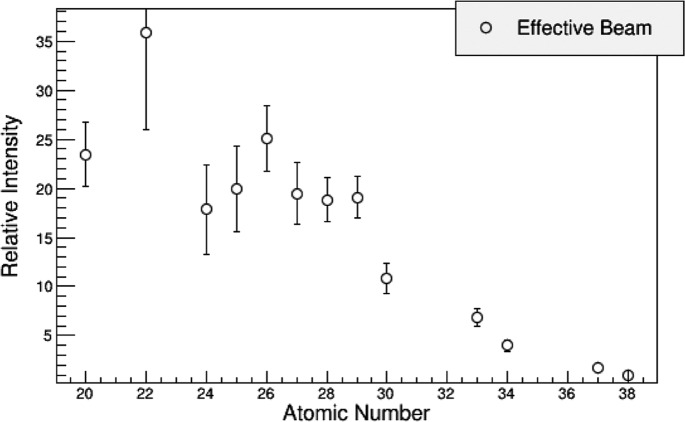
Effective beam for SRM 610. Intensities are given relative to that of the Sr *K*α line.

To calibrate the effective beam, the element concentrations as given by NIST are treated as known constants in equation (1). The energy *E*_0_ is set to 18995 eV as only one of the beam energy or the incident intensity needs to be varied in this equation in order to construct the effective beam. This value was chosen such that the cross sections (σ is the total interaction cross section and τ is the photo-absorption cross section) of the various elements will be similar and at the same time the approximation 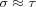 is valid (Henke *et al.*, 1993 ▶). In order to construct the effective beam, iterative adjustments are applied to 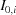 such that
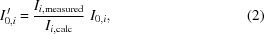
where 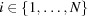. 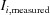 and 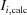 are the measured corrected peak intensity and the intensity determined using equation (1), respectively. The result is a set of *N* monochromatic beams for the elements detected in the sample. Fig. 1 ▶ shows the spectrum obtained for SRM 610.

The elements in Fig. 1 ▶ are a subset of the elements in the sample. They are elements with defined concentrations and with *K*α emission lines in the detectable range. The method, at this level, could just as well be applied to other shells.

Differences between samples are expected in that the spectrum ‘seen’ by the sample depends on its composition. The effective beam is compensating for this spectrum and must vary to some extent with sample composition. For this reason, the best results are expected when the calibration standard and analytes are similar in their absorption/enhancement properties. Preliminary measurements with other references suggest that the general spectrum shape varies little when derived from a variety of natural minerals such as apatite, clinopyroxene and biotite. However, the errors associated with those measurements are large due to the low statistics collected, and the results are not conclusive enough to be used in the construction of an effective beam. For this reason we have opted to not include them in this paper. On the other hand, given the fact that the effective beam constructed with data collected with SRM 610 successfully determined the concentrations of elements in natural minerals, as discussed in §4.5, it follows that an effective beam determined by spectra from those same minerals should give an effective beam like that obtained with SRM 610.

### 4.2. Effective beam functional form   

As a general trend appears in the effective beam spectrum, it is reasonable to fit it with a functional form for application to all detectable elements. Only *K*-shell emissions are considered. A functional form can be obtained by considering the detected intensity in the first approximation (primary excitation only). It is further assumed that the incident spectrum can be described by a normalized distribution 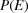 and constant intensity 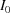 over the incident photon energy range 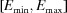. The detected intensity of line *i* is then
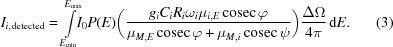
All other quantities are as defined above. The monochromatic effective beam, 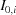, for element *i* is related to the detected intensity, 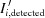, by
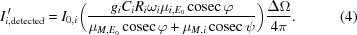
The intensity of the effective beam must be such that 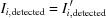. Equating equation (3) and equation (4) and cancelling terms as appropriate gives
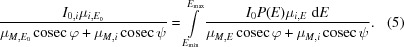
The mass absorption coefficients are taken to have the form given by Heitler (1954 ▶) and are shown in equation (6). Taking *N* to be the concentration of absorbers in the material, where 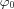 is the Thomson scattering cross section, *m* is the mass of an electron and *k* is the photon energy, this has the form
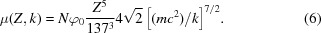
After substitution into equation (5), solving for 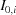 and cancelling terms, the result is
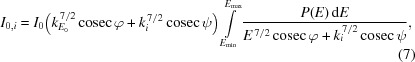
where *E* is used to denote the energies of the incident photon spectrum, and *k* is the energies of other photons. For the case at hand 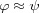, allowing the simplification of these terms, though they can be retained in the general case.

**Figure 2:**
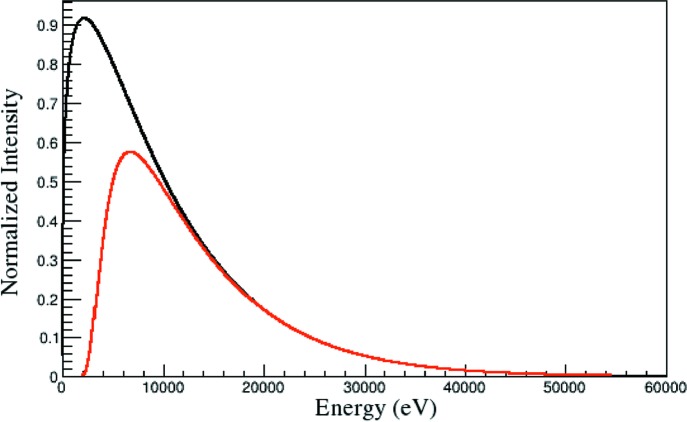
Synchrotron radiation spectra as described by equation (8). The black curve shows the original spectrum. The red curve shows the spectrum after attenuation by 10 cm of air (Henke *et al.*, 1993 ▶).

It is expected that the form of 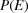 should be related to the general synchrotron radiation spectrum for a bending magnet, which is described by Jackson (1999 ▶). The relevant result given by Jackson (1999 ▶), where 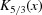 is a modified Bessel function, is that
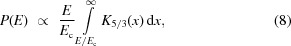
where constants can be ignored and *E*_c_ is the critical energy of the synchrotron. This result gives the standard form for synchrotron radiation shown in Fig. 2 ▶.

### 4.3. Accounting for beam attenuation   

**Figure 3:**
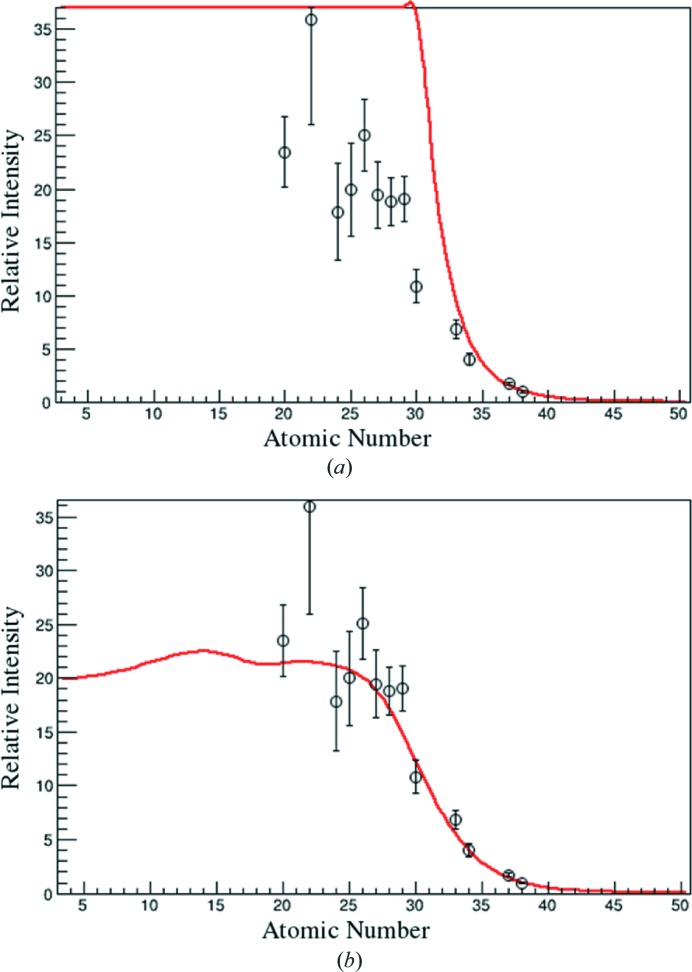
Functional description of effective beam. Relative intensity *versus* atomic number effective beam spectra with and without attenuation of the incident spectrum. Based on the result in equation (10). The functional form is in red, data are shown with their uncertainties. Attenuation (*b*) increases the minimum energy of the spectrum, which introduces a ‘kink’ in the effective beam spectrum. The non-attenuated result (*a*) captures the general trend of the data, but not its details.

Another feature that must be incorporated into the description of the spectrum is that the spectrum given in equation (8) can be attenuated by any matter encountered by the beam prior to and in the sample. In particular, the beamline optics, ion chambers, filters and any air or other media that must be traversed. All of these features are present at VESPERS. To account for the attenuation of the beam a factor 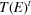 is used. 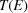 is taken to be the transmission of X-rays by 10 cm of air as a function of the energy, as given by Henke *et al.* (1993 ▶). The choice of attenuation by 10 cm is arbitrary. It serves simply to provide an appropriate functional form for describing the attenuation of the beam. This function can be chosen as convenient; only the power *t* will change in the final fitting. The power *t* will be a parameter that adjusts the degree of attenuation. The effect of the transmission coefficient is shown in Fig. 2 ▶. Attenuation by air and optics on the beamline shifts the minimum energy of the spectrum. Without this shift, the observed form of the effective beam is not achieved, as shown in Fig. 3 ▶.

**Figure 4:**
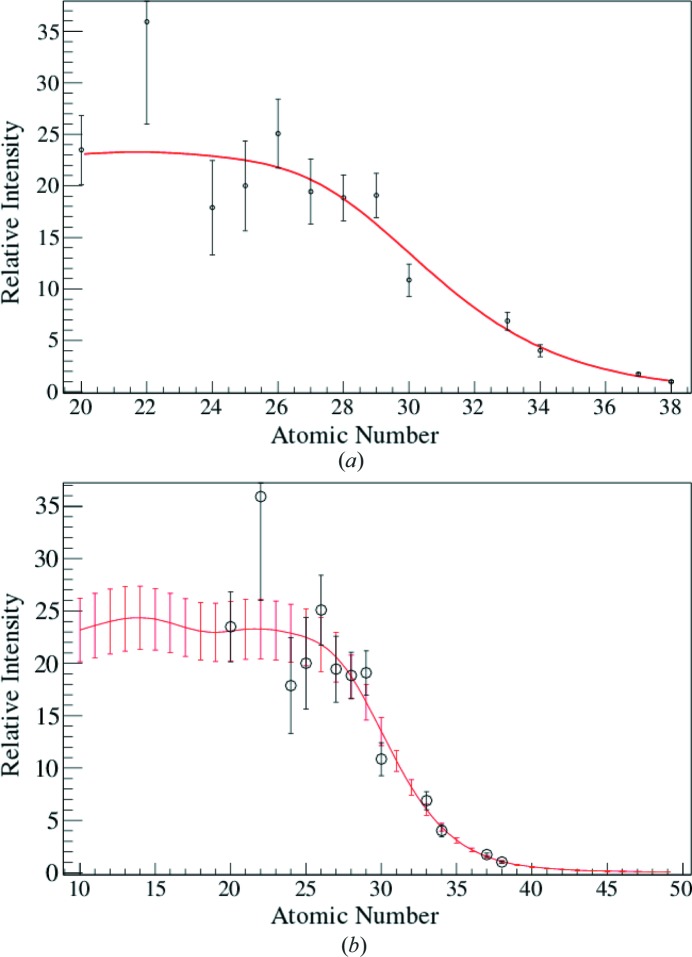
(*a*) Fit (in red) to the effective beam data with equation (10) and (*b*) effective beams determined by fit results (in red), with their errors and compared with the data.

Putting equation (8) and the attenuation factor into equation (7) gives
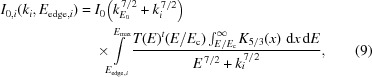
where it is used that for a given element *i* the value of 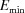 will always equal that of the absorption edge, 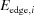. The powers of the photon energies are taken to be parameters, as is the leading coefficient 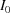. The former helps adjust for deviations of the exponents of the energy terms from 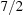, which is expected from the discussion in the work by Heitler (1954 ▶). It is also used that the photon emission and absorption edge energies, as given in Henke *et al.* (1993 ▶), can be described by nearly quadratic functions of the atomic number *Z*. The parameters for these functions, denoted *a*, *b*, *c* and *d*, are determined by a least-squares fit and fixed. This gives the final fitting function
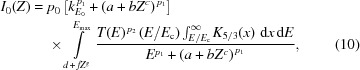
with free parameters 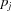, 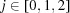. The result of the fit is shown in Fig. 4 ▶.

The corresponding parameter values are given in Table 1 ▶. The value of the intensity parameter (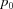) is of little consequence, as its effect disappears in the process of concentration calculations. The power of the attenuation function (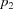) may be indicative of the amount of material in the path of the incident beam, but ultimately ensures an appropriate minimum energy for the incident beam. The parameter value in Table 1 ▶ gives a minimum beam energy in the range of the supported value of 6 keV (Feng *et al.*, 2007 ▶). The power of the energy terms is double that expected from the basic description of photo-absorption given by Heitler (1954 ▶). Some deviation is expected, however, because this description is somewhat idealized. The effect is also mitigated by the appearance of similar terms in the numerator and denominator of equation (10).

### 4.4. Element concentrations – reference sample   

**Figure 5:**
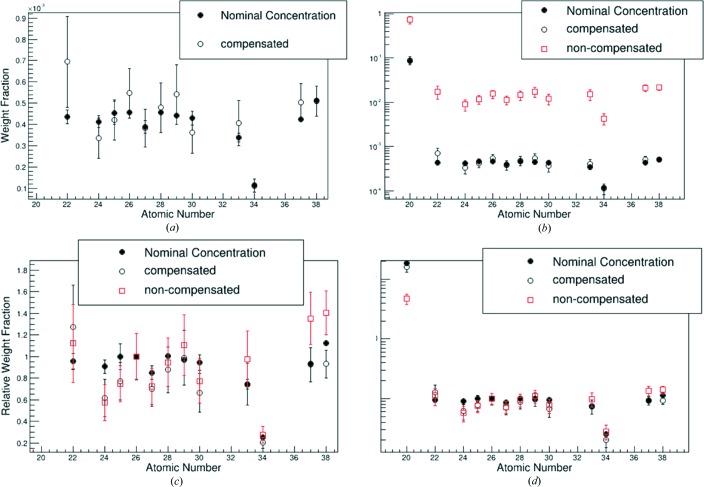
Application of the functional effective beam and fundamental parameters method to SRM 610 data. Both ‘compensated’ (include undetected elements from SRM 610) and ‘non-compensated’ (detected elements only) results are shown. (*a*) Absolute concentrations of results. (*b*) Log-scale of absolute concentration plot. Compensated and nominal values only. (*c*) Concentrations relative to *C*_Fe_. (*d*) Same as the bottom left, but using log-scale.

It must also be asked whether the concentrations for the SRM 610 sample are reproduced if the effective beam is used for an iterative concentration determination with equation (1). The concentrations are calculated using the previously fitted beam profile, as discussed in the previous section. The results of the determination and a comparison with the concentrations quoted by NIST are shown in Fig. 5 ▶.

There are two variations of the calculation shown in the figure. One compensates for the undetected elements in the sample by including them in the mass absorption coefficient of the matrix and the normalization of the concentrations. A reference element is chosen and all undetected elements are included in their nominal concentrations relative to the reference element. The other uses only detected elements in the calculation. The latter case is relevant, as in general the presence of elements is only known if they are detected.

Looking at the absolute concentrations in Fig. 5 ▶, it can be seen that the non-compensated and nominal values follow the same general trend, though they are shifted relative to each other. The same can be said for the non-compensated values. The discrepancy is due to normalization of the values. This is eliminated by looking at the relative concentrations. In the same figure, the concentrations are given relative to the iron concentration. The determined values are no longer shifted with respect to the nominal values. It can also be seen that both the compensated and non-compensated results agree with the nominal concentrations over much of the range. There are a still a few deviations, which may be due, in part, to small-scale variations in the SRM 610 sample.

It is critical that there is agreement between the non-compensated and nominal concentrations as the non-compensated scenario is the one that matches the general experimental conditions. If agreement is reached here, then it is anticipated that the relative concentration values will be reliable for other samples where some fraction of the elements in the matrix are unknown.

It can be seen in Fig. 5 ▶ that, if the data sets are normalized to the iron concentration, the non-compensated and nominal concentrations agree in most instances. Agreement with the nominal values is better with the non-compensated results than the compensated results, in some cases. This could be expected on the grounds that the compensation method enforces the assumption that a series of elements in the sample are present in certain amounts. All results will be influenced by the validity of this assumption, as it alters the absorption enhancement effects in the sample. Ca, Rb and Sr show the greatest disagreement with their respective nominal values. The Ca concentration may be prone to variation on the scale used here, as it is just a component of the glass matrix. Nonetheless, the compensated determined concentration for Ca is similar to the nominal value. Rb and Sr take on the nominal trend for the non-compensated calculation. This suggests that for this pair the true absorption and enhancement effects are better accounted for when no assumptions about the matrix composition are enforced.

### 4.5. Element concentration – natural minerals   

Data have been collected for several naturally sourced mineral references. Two considered here are clinopyroxene [Ca(MgFe)Si_2_O_6_] and biotite [K(Mg,Fe)_3_(AlSi_3_O_10_)(OH)_2_]. Concentrations referred to will be atomic fractions, whether discussing nominal or determined values. Because these are natural samples, they contain many trace elements in addition to the nominal ones. Deviations from nominal concentrations are also possible. Only the nominal elements will be considered, because, to test the determination of trace elements, measurements through another method must be performed. The nominal ratio *C*_Fe_/*C*_K_ = 3 is expected for biotite. The nominal ratio *C*_Fe_/*C*_Ca_ = 1 is expected for clinopyroxene. X-ray fluorescence measurements on the biotite reference give non-compensated concentrations *C*_K_ = 0.28 ± 0.08 and *C*_Fe_ = 0.62 ± 0.10 and a ratio of *C*_Fe_/*C*_K_ = 2.2 ± 0.8. Similarly for the clinopyroxene sample it was determined that *C*_Ca_ = 0.51 ± 0.12 and *C*_Fe_ = 0.41 ± 0.07, yielding a ratio *C*_Fe_/*C*_Ca_ = 0.80 ± 0.2. Both of these results agree with expectations from the nominal mineral compositions. As with the SRM 610 sample, the relative concentrations agree with the nominal values without considering undetected elements. It should also be noted that these samples all represent different matrices, suggesting validity of the method for different sample types. The matrices of SRM 610 and these minerals are similar in that a considerable portion of each material is low-*Z* elements, which is typical of many minerals. In the event that all elements can be detected, the absolute concentrations should be valid estimates of the real concentrations and accuracy of results should improve. The precision of the concentrations presented here is largely constrained by the uncertainty in fitting the peaks in the collected XRF spectra. Reduction in the uncertainty can be achieved through improved statistics and fitting methods (Solé *et al.*, 2007 ▶).

It is also interesting to note that the clinopyroxene data and biotite data were taken during different runs on the VEPSERS beamline. The biotite and SRM 610 data were taken during the same run. Further, the two runs used different XRF detectors. It can be suggested that the effective beam results can be applied across runs, assuming that no major change in beamline conditions has occurred that would greatly effect the beam spectrum.

## 5. Conclusions   

Evidence has been given supporting that the effective beam method, in the form given herein, provides an accurate means to assess the concentration of elements in XRF spectra, taken from polyatomic samples, using a polychromatic incident spectrum. Though presented for synchrotron data, it should be useful for other sources. The semi-physical function used here could be remodelled for other sources, or a simple polynomial fit used.

Further definition and testing of the effective beam spectrum given here can be performed through the use of additional standards. Improvements in the accuracy and precision of the results are expected with the use of well defined references on the scale of measurement and higher statistics data, respectively.

## Figures and Tables

**Table 1:** Parameter values for fit to effective beam data with equation (10)

Parameter, *p*	Value	Error
0	0.00101	0.00008
1	7.0	0.1
2	41	2
